# Analysis of Microcirculation Changes in the Macular Area and Para-Optic Disk Region After Implantable Collamer Lens Implantation in Patients With High Myopia

**DOI:** 10.3389/fnins.2022.867463

**Published:** 2022-05-19

**Authors:** Yingnan Xu, Weihua Yang, Tan Long, Weihong Shang, Xiangzhong Xu, Jinfan Wang, Jin Yao, Keran Li

**Affiliations:** ^1^Department of Ophthalmology, The Affiliated Eye Hospital of Nanjing Medical University, Nanjing, China; ^2^Department of Ophthalmology, Xi’an No. 1 Hospital, Xi’an, China; ^3^School of Marxism, Nanjing Medical University, Nanjing, China

**Keywords:** blood flow density, implantable collamer lens implantation, optical coherence tomography angiography, macular area, myopia, para-optic disk, retinal microcirculation

## Abstract

Myopia has become an important public health problem to be solved urgently. Posterior chamber phakic implantable Collamer lens (ICL) implantation is one of the latest and safest products for myopia correction worldwide. This prospective cross-sectional case series aimed to observe changes in the macular retinal thickness, retinal nerve fiber layer (RNFL) thickness of para-optic disk region, and blood flow density after posterior ICL implantation in patients with high myopia using optical coherence tomography angiography (OCTA). A total of 67 eyes of 67 patients with high myopia, who underwent ICL implantation at The Affiliated Eye Hospital of Nanjing Medical University from January 2020 and December 2020, were included. The spherical equivalent (SE) of the operative eyes was >−6.00 D. The changes in vision, intraocular pressure (IOP), SE, and vault were observed pre-operatively, and follow-up were performed 1 week, 1 month, and 3 months. OCTA was used to observe the changes in the CRT, retinal thickness of paracentral fovea, FAZ, superficial and deep retinal blood flow density in the macular area, RNFL thickness of para-optic disk region, and blood flow density before and after ICL implantation. The uncorrected distance visual acuity (UDVA) and best corrected distance visual acuity (CDVA) of the patients post-operation were significantly improved (*P* < 0.001). The IOP increased in comparison with other time points at 1 week post-operation (*P* < 0.05). There were no significant changes in CRT post-operation. The retinal thickness in the upper, lower, nasal, and temporal quadrants of the paracentral fovea increased significantly at 1 month and 3 months post-operation (*P* < 0.05). The FAZ area at all postoperative time points were decreased (*P* < 0.001). At 3 months post-operation, the blood flow density of the superficial and deep retinal layers in the upper, lower, and nasal macular area were significantly reduced (*P* < 0.05). At 1 month post-operation, the RNFL thickness in the temporal para-optic disk region and blood flow density were significantly reduced (*P* = 0.001 and *P* < 0.05, respectively). ICL implantation for highly myopic eyes led to an increase of the retinal thickness in the upper, lower, nasal, and temporal regions of the paracentral fovea; reduction of RNFL thickness in the temporal area of para-optic disk; decrease in FAZ area; and decrease in the blood flow density of some deep and superficial retinal layers as well as that of the temporal para-optic disk region.

## Introduction

Posterior chamber phakic implantable collamer lens (ICL) implantation has become one of the most widely used surgical methods to correct high myopia worldwide, and its effectiveness and safety have been validated by long-term investigations and studies (Packer, 2018). V4C is a new type of ICL, which is improved on the basis of conventional ICL that reduces the incidence of pigment disseminated (pigmentary) glaucoma and complications such as subcapsular cataract (Fernandes et al., 2011). Previous studies have shown that the uncorrected distance visual acuity (UDVA) and best corrected distance visual acuity (CDVA) can reach or be better than 0 logMAR after ICL implantation. Even if the post-operative UDVA and CDVA were lower than 0 logMAR, they were still significantly higher than the pre-operative CDVA (Fernandes et al., 2011).

Studies have shown that in people with high myopia, the axis of the eye grows, the retina becomes thinner, oxygen consumption increases, and the compensatory effect of blood oxygen saturation of retinal blood vessels disappears, which easily lead to microvascular changes. In addition, the increase of eye axis will also lead to the decrease of choroidal blood flow and the thinning of choroidal thickness, further leading to the decrease of blood perfusion in deep capillary network, which is also an important reason why high myopia is more likely to develop into pathological myopia (de Carlo et al., 2016; Ye et al., 2020). However, the residual viscoelastic agent during ICL surgery can lead to elevated intraocular pressure (IOP) in patients with high myopia, while peak IOP significantly reduced intraocular blood flow (Chen et al., 2019). Furthermore, the destruction of the homeostasis of the ocular environment and release of inflammatory factors during intraocular surgery significantly increased the incidence of cystoid macular edema, which further affected retinal perfusion (Han et al., 2019). Optical Coherence tomography angiography (OCTA) is a fast, high-resolution and non-invasive fundus angiography technique. It can be used to measure the retinal capillary plexus at different depths around the fovea, quantify the Macular vascular density (MVD) and Foveal avascular zone (FAZ) of different retinal layers (Gella et al., 2011). Therefore, OCTA examination was performed on patients diagnosed with high myopia and undergoing ICL surgery, in order to more comprehensively evaluate postoperative retinal circulation parameters and explore the influence of their changes on the occurrence and development of pathological myopia.

## Materials and Methods

### Participants

This was a prospective cross-sectional study that included patients who were diagnosed with high myopia and underwent ICL implantation at The Affiliated Eye Hospital of Nanjing Medical University from January 2020 and December 2020. The study complied with the Declaration of Helsinki and was approved by the Medical Ethics Committee of The Affiliated Eye Hospital of Nanjing Medical University (Batch Number: 2019007). All patients or their guardians had signed a form of informed consent before the surgery.

The inclusion criteria were as follows: (1) The surgical eye with spherical equivalent (SE) >−6.00 D; (2) patient was willing to improve the refractive status through ICL surgery; (3) the refractive power was relatively stable (the annual change in refractive power for two consecutive years was ≤0.50 D); (4) central anterior chamber depth ≥2.8 mm, opened corner; (5) corneal endothelial cell density ≥2,000 cells/mm^2^; (6) stable corneal shape; (7) no progressive lens opacities; and (8) patients who had normal retina or retinal tears have been treated with laser photocoagulation.

The exclusion criteria were as follows: (1) Pathological myopia, including macular choroidal neovascularization, lacquer crack, choroidal retinal atrophy, macular retinal split and hole, posterior scleral staphyloma, high myopia optic neuropathy, etc.; (2) keratoconus, severe dry eye, active eye disease, or infection; (3) patients with fundus diseases such as uncontrolled glaucoma, cataracts that seriously affect vision, and retinal detachment; (4) patients with systemic organic diseases such as systemic connective tissue diseases or autoimmune diseases that affect surgical recovery; (5) history of eye surgery or trauma; (6) individuals with mental or psychological abnormalities, or those who were unable to understand the surgical risks or had unrealistic expectations; (7) patients who are unable to follow-up regularly; (8) IOP > 21 mmHg 1 day after the surgery; (9) patients with ICL adjustment during the follow-up period.

### Examination Equipment

#### Routine Examinations

All patients underwent routine examinations before surgery, including those for UDVA and CDVA measurement, subjective and objective optometry, IOP (Topcon, Japan), slit lamp microscope and indirect ophthalmoscope fundus examination, Pentacam (OCULUS, Wetzlar, Germany), axial measurement (IOLMaster Zeiss 500; Carl Zeiss Meditec, Jena, Germany), vault and corneal endothelial cell density measurement. All pre-operative examinations were performed by designated permanent professionals and optometrists.

#### Imaging Examinations

**FIGURE 1:**
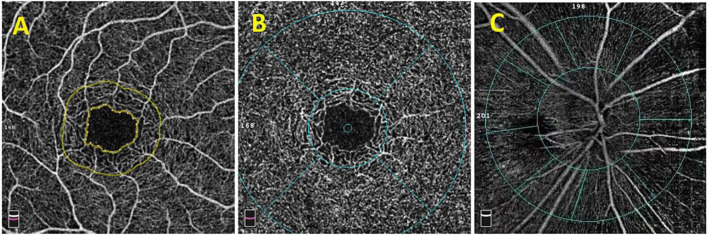
OCTA image of the macular area and optic disk. **(A)** The macular avascular area. **(B)** The fovea (the range within the inner ring) and the paracentral fovea (the range between the inner and outer rings). The diameter of the inner ring is 1.0 mm, and the diameter of the outer ring is 3.0 mm. The macular fovea is divided into upper, lower, nose, and temporal quadrants. **(C)** Images of para-optic disk (the range between the inner ring and the outer ring). It is divided into four quadrants: upper, lower, nose, and temporal.

The scanning speed of OCTA (RTVue XR Avanti, Optovue Inc., Fremont, CA, United States) was 70000 A-Scans per second, the wavelength of the light source was 840 nm, and the bandwidth was 50 nm. Each OCTA image was composed of 304 pixels in the horizontal and vertical directions. Images with signal parameters >40 were included in the study. The range of the scanning region spanned from the inner limiting membrane (ILM) to the retinal pigment epithelium layers. The macular fovea was defined as an annular area of 1 mm in the center of the macula, and the paracentral fovea was defined as a round ring with an outer diameter of 3 mm and inner diameter of >1 mm located at the center of the macula. Retinal thickness referred to the average thickness of a specific area, and full retinal thickness referred to the distance from the ILM to the retinal pigment epithelium–Bruch’s membrane complex. Regarding the measurement of macular retinal blood vessel density, we used OCTA images to analyze the deep and superficial layers of the retina. The superficial retina was defined as the retina from 3 μm under the ILM to 15 μm under the inner plexiform layer (IPL), and the deep retina was defined as the retina from 15 to 17 μm under the IPL. The optic disk boundary was delineated along the optic canal opening, and the para-optic region was defined as an oval ring extending 0.7 mm from the optic disk boundary and the perioptic disk region was divided into four quadrants: upper, lower, nasal, and temporal. The blood vessel density was defined as the percentage of the area occupied by blood vessels and capillaries within the observation range. In order to ensure the consistency and accuracy of the test results, all the OCTA examinations were completed by the same experienced physician and the results have been automatically corrected by the equipment software before calculating data. All the values were automatically obtained and calculated by the instrument and the each signal value was >Q5 (Figure 1).

### Surgical Procedure and Post-operative Medication

A 3.2-mm clear corneal incision was made at the scleral margin of the temporal limbus of the eye, and an auxiliary corneal incision was made at the 12 o’clock position. After injection of viscoelastic agent into the anterior chamber, an ICL was implanted and then ICL adjustment was performed. Four loops were placed in the posterior ciliary sulcus of the iris. The astigmatism correction type ICL was rotated to the required axial position according to the navigation and verified and centered the position. Manual I/A was used to aspirate viscoelastic agent completely. All operations were performed by the same experienced surgeon.

After surgery, 0.5% levofloxacin eye drops (Santen Pharmaceutical Co., Ltd., Osaka, Japan) were administered four times daily for 1 month; 0.1% bromfenac sodium eye drops (Jiangsu Chengxin Pharmaceutical Co., Ltd., Japan) three times daily for 2 weeks; 0.3% sodium hyaluronate eye drops (Santen Pharmaceutical Co., Ltd., Osaka, Japan) three times daily for 1 month; and prednisolone acetate eye drops (Allergan, Westport, Ireland) three times daily, decreasing once a week for a total of 3 weeks. No intra-operative and post-operative complications occurred.

### Post-operative Follow-Ups

Follow-up were performed 1 week, 1 month, and 3 months after operation. All relevant parameters were collected, except for the pre-operative axial examination.

### Statistical Methods

All statistical analysis were performed with SPSS 22.0 software. All data were expressed as the mean ± standard deviation, and a population analysis was performed using the mean values. Repeated measures analysis of variance was performed on all data, and the Bonferroni correction was used for pairwise comparisons. *P* < 0.05 was considered to be statistically significant.

## Results

### Overall Conditions

A total of 67 patients (67 eyes, right eyes) with high myopia who underwent ICL implantation were included in this study for analysis. There were 34 males (50.75%) aged 25.86 ± 5.01 years, and 33 females (49.25%) aged 25.9 ± 5.3 years. The patients’ pre-operative axial length was 27.24 ± 1.23 mm, anterior chamber depth was 3.23 ± 0.25 mm, and horizontal corneal diameter (white-to-white) was 11.55 ± 0.38 mm. A total of three cases were excluded during the study and follow-up. Among them, one had IOP higher >21 mmHg on the first day after surgery and was treated with IOP-lowering drugs. One patient’s vault was >1500 μm, and after the ICL was adjusted to vertical position, the vault was reduced to 980 μm. Finally, one patient was lost to follow-up after surgery.

### Pre-operative and Post-operative Changes in Visual Acuity, Spherical Equivalent, and Intraocular Pressure

The overall difference in LogMAR UDVA before surgery and at 1 week, 1 month, and 3 months after surgery was statistically significant (*F* = 838.697, *P* < 0.001). The UDVA at each time points after surgery were significantly higher than that before surgery, and the differences were all statistically significant (*P* < 0.001). However, there was no statistically significant difference in UDVA at all follow-up time points (*P* > 0.5). The overall difference in LogMAR CDVA before surgery and at 1 week, 1 month, and 3 months after surgery was statistically significant (*F* = 23.582, *P* < 0.001). Furthermore, the CDVA at each time point after surgery was significantly improved (*P* < 0.05). The SE at each time point post-surgery was significantly lower than that pre-operatively (*F* = 1381.582, *P* < 0.001). Overall comparison of the IOP before and at 1 week, 1 month, and 3 months after the surgery showed that the differences were statistically significant (*F* = 10.683, *P* = 0.000). Further pairwise analysis showed that the IOP increased at 1 week post-operatively, and the difference was statistically significant (*P* < 0.05). The IOP reached the pre-operative baseline level at 1 month after surgery (*P* > 0.5). The results are shown in Table 1.

**TABLE 1:** Comparison of general conditions of patients before and after surgery.

	Preoperative	1 week after surgery	1 month after surgery	3 months after surgery	*F*	*P*
IOP	15.692.41	17.012.48*^ade^*	15.662.65	15.782.07	10.683	0.000
SE	−9.732.14*^abc^*	−0.210.31	−0.190.24	−0.180.25	1381.582	0.000
UDVA	1.410.40*^abc^*	0.030.07	0.020.06	0.010.05	838.697	0.000
CDVA	0.010.03*^abc^*	−0.020.05	−0.020.05	−0.050.06*^ef^*	23.582	0.000
Vault	−	568.06209.65*^de^*	543.07204.56	530.39201.54	15.459	0.000

*IOP, intraocular pressure; SE, spherical equivalent; UDVA, uncorrected distance visual acuity; CDVA, corrected distance visual acuity. ^a^P < 0.05, values compared before operation and 1 week after surgery.*

*^b^P < 0.05, values compared before surgery and 1 month after surgery.*

*^c^P < 0.05, values compared before surgery and 3 months after surgery.*

*^d^P < 0.05, values compared 1 week after surgery and 1 month after surgery.*

*^e^P < 0.05, values compared 1 week after surgery and 3 months after surgery.*

*^f^P < 0.05, values compared 1 month after surgery and 3 months after surgery.*

### Post-operative Vault

The vault was normal at each time point in the post-operative follow-up period, and it gradually decreased with time (*F* = 15.459, *P* < 0.001). The results are shown in Table 1.

### Retinal Thickness Changes in the Macula and Paracentral Fovea

**FIGURE 2:**
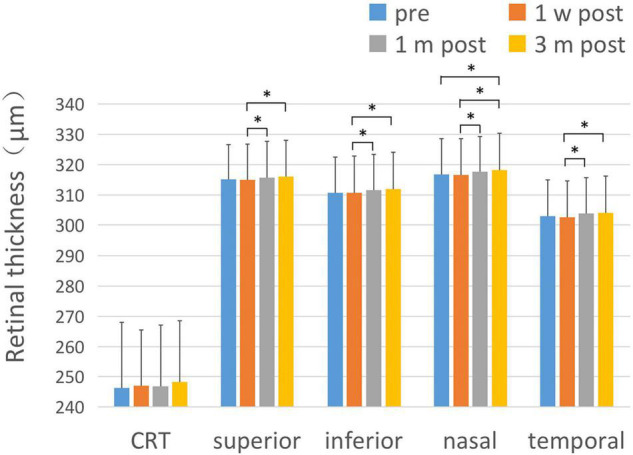
Changes of retinal thickness with respect to CRT and the parafoveal area. After ICL implantation, the changes of retinal thickness with respect to CRT and the paracentral fovea area in each quadrant were compared. There was no significant change in CRT after ICL implantation compared with pre-operative values. The thickness of the retinal area of paracentral fovea in each quadrant at 1 month and 3 months after the operation was significantly thicker than that at 1 week after the operation, and the retinal thickness in the nasal paracentral fovea area was significantly thicker 3 months after the operation compared to pre-operative values. The short black line shows that the data at the corresponding two time points were statistically different. *P < 0.05.

There was no statistically significant difference of central retinal thickness (CRT) at each time point before and after the surgery (*F* = 0.856, *P* = 0.465). There were statistically significant differences in the retinal thickness of the paracentral fovea in the four quadrants of the upper, lower, nasal, and temporal sides before and after surgery at each time point (F1 = 3.182, P1 < 0.05; F2 = 4.845, P2 < 0.01; F3 = 7.368, P3 < 0.001; F4 = 7.464, P4 < 0.001). 1 month and 3 months after surgery, the retinal thickness of the paracentral fovea in each quadrant was significantly thicker than that at 1 week after surgery. 3 months after the operation, the retinal thickness of the paracentral fovea in the nasal side was significantly thicker than that before surgery. The results are shown in Figure 2 and Table 2.

**TABLE 2:** Changes of retinal thickness in macular area at each time point.

	Preoperative	1 week after surgery	1 month after surgery	3 months after surgery	*F*	*P*
CRT	246.2521.68	247.1018.37	246.93 ± 20.20	248.2520.19	0.856	0.465
Upper	315.1011.56	315.00 ± 11.73*^de^*	315.7211.86	315.9412.03	3.182	0.033
Lower	310.6411.89	310.67 ± 12.18*^de^*	311.5711.84	311.8812.21	4.845	0.003
Nasal	316.7011.78*^c^*	316.6012.00*^de^*	317.5511.75	318.1212.19	7.368	0.000
Temporal	302.9611.96	302.5512.05	303.7611.89	303.9412.31	7.464	0.000

*CRT, central retinal thickness; upper, the upper quadrant of the paracentral fovea; lower, the lower quadrant of the paracentral fovea; nasal, the nasal quadrant of the paracentral fovea; temporal, the temporal quadrant of the paracentral fovea. ^c^P < 0.05, values compared before surgery and 3 months after surgery. ^d^P < 0.05, values compared 1 week after surgery and 1 month after surgery. ^e^P < 0.05, values compared 1 week after surgery and 3 months after surgery.*

### Foveal Avascular Zone Area

**FIGURE 3:**
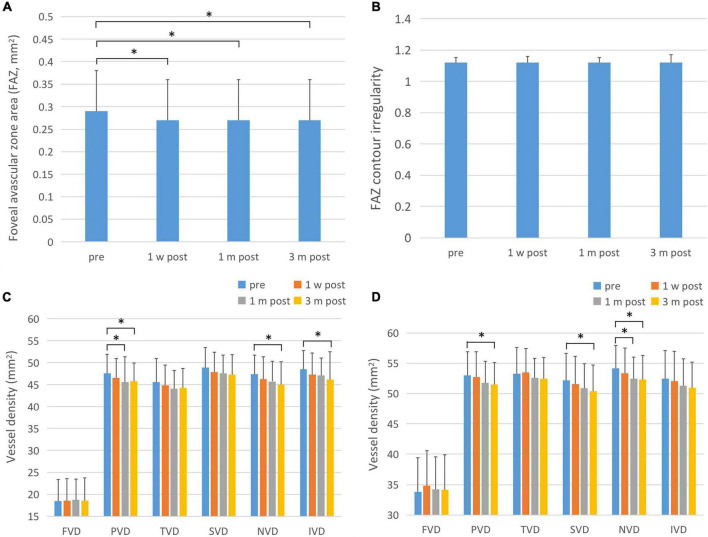
FAZ-A, FAZ-CI, and blood flow changes in the fovea and parafoveal area after ICL implantation. **(A)** The changes in FAZ-A after operation. The FAZ-A values at various time points after surgery were significantly lower than those before surgery. **(B)** Post-operative FAZ-CI changes, showing that there is no statistical difference between post-operative and pre-operative values. **(C)** Changes in superficial retinal blood flow after surgery. The PVD, NVD, and IVD were significantly reduced 3 months after surgery. One month after surgery, PVD was also significantly reduced compared to pre-operative values. **(D)** Postoperative changes of deep retinal blood flow. The PVD, SVD, and NVD were significantly reduced at 3 months after surgery, and NVD was significantly decreased 1 month after surgery. The short black line shows that the data at the corresponding two time points were statistically different. *P < 0.05.

There was a statistically significant difference in the overall FAZ area (FAZ-A) at each time point before and after surgery (*F* = 36.503, *P* < 0.001). The FAZ area at each time point post-surgery was lower than that pre-operatively (*P* < 0.001). The comparison of FAZ contour irregularity (FAZ-CI) at various time points before and after surgery showed no significant difference (*F* = 0.720, *P* = 0.541). The results are shown in Figure 3 and Table 3.

**TABLE 3:** Changes of macular blood flow density at each time point.

	Preoperative	1 week after surgery	1 month after surgery	3 months after surgery	*F*	*P*
FAZ area	0.290.09*^abc^*	0.270.09	0.270.09	0.270.09	36.503	0.000
FAZ contour	1.120.03	1.120.04	1.120.03	1.120.05	0.720	0.541
Superficial FVD	18.474.95	18.644.98	18.744.75	18.635.17	0.151	0.929
Superficial PVD	47.594.28*^bc^*	46.554.43	45.605.71	45.784.15	3.418	0.018
Superficial TVD	45.605.33	44.854.61	44.054.17	44.314.40	2.119	0.099
Superficial SVD	48.934.52	47.904.51	47.584.10	47.334.45	2.694	0.052
Superficial NVD	47.404.36*^c^*	46.235.09	45.734.60	44.985.19	4.409	0.005
Superficial IVD	48.474.27*^c^*	47.304.86	47.064.04	46.136.37	3.555	0.015
Deep FVD	33.825.61	34.825.77	34.235.34	34.185.74	2.711	0.055
Deep PVD	53.023.89*^c^*	52.724.15	51.803.51	51.513.59	3.458	0.017
Deep TVD	53.254.30	53.493.95	52.573.23	52.493.42	1.801	0.148
Deep SVD	52.204.43*^c^*	51.594.57	50.874.03	50.404.30	2.968	0.033
Deep NVD	54.193.71*^bc^*	53.324.17	52.493.55	52.343.96	4.914	0.003
Deep IVD	52.474.64	52.044.95	51.294.46	50.974.21	1.983	0.118

*FAZ, foveal avascular zone; FVD, fovea vessel density; PVD, para-fovea vessel density; mo, month; TVD, temporal vessel density; SVD, superior vessel density; NVD, nasal vessel density; IVD, inferior vessel density. ^a^P < 0.05, values compared before operation and 1 week after surgery.*

*^b^P < 0.05, values compared before surgery and 1 month after surgery.*

*^c^P < 0.05, values compared before surgery and 3 months after surgery.*

### Macular Blood Flow Density

There was no statistically significant difference in blood flow density of superficial macular fovea, temporal and upper paracentral fovea at each time point before and after surgery (F1 = 0.151, P1 = 0.929; F2 = 2.119, P2 = 0.099; F3 = 2.694, P3 = 0.052). There were statistically significant differences in blood flow density of superficial paracentral fovea at each time point before and after surgery (*F* = 3.418, *P* = 0.018), among which, 1 month and 3 months after surgery were significantly lower than those before surgery (P1 = 0.035, P2 = 0.029). The difference in blood flow density of the superficial nasal side and inferior paracentral fovea at each time point before and after surgery was statistically significant (F1 = 4.409, P1 = 0.005; F2 = 3.555, P2 = 0.015), which significantly decreased 3 months after surgery compared with that pre-operatively (P1 = 0.000; P2 = 0.047). The results are shown in Figure 3 and Table 3.

There was no statistically significant difference in blood flow density in the deep fovea, temporal, and inferior paracentral fovea areas before and after surgery (F1 = 2.711, P1 = 0.055; F2 = 1.801, P2 = 0.148; F3 = 1.983, P3 = 0.118). There were statistically significant differences in the blood flow density of deep paracentral fovea at each time point before and after surgery (*F* = 3.458, *P* = 0.017), among which it was significantly lower at 1 month and 3 months after surgery than that pre-operatively (P1 = 0.035, P2 = 0.029). The difference in blood flow density of the deep layer above the paracentral fovea at different time points was statistically significant (*F* = 2.968, *P* = 0.033), and it was significantly decreased 3 months after surgery compared with that pre-operatively (*P* = 0.012). There were statistically significant differences in the blood flow density of the deep nasal paracentral fovea at different time points (*F* = 4.914, *P* = 0.003), and it was significantly lower at 1 month and 3 months after surgery than that pre-operatively (P1 = 0.003, P2 = 0.020). The results are shown in Figure 3 and Table 3.

### Para-Disk Nerve Fiber Layer Thickness and the Blood Flow Density of Radial Peripapillary Capillaries

**FIGURE 4:**
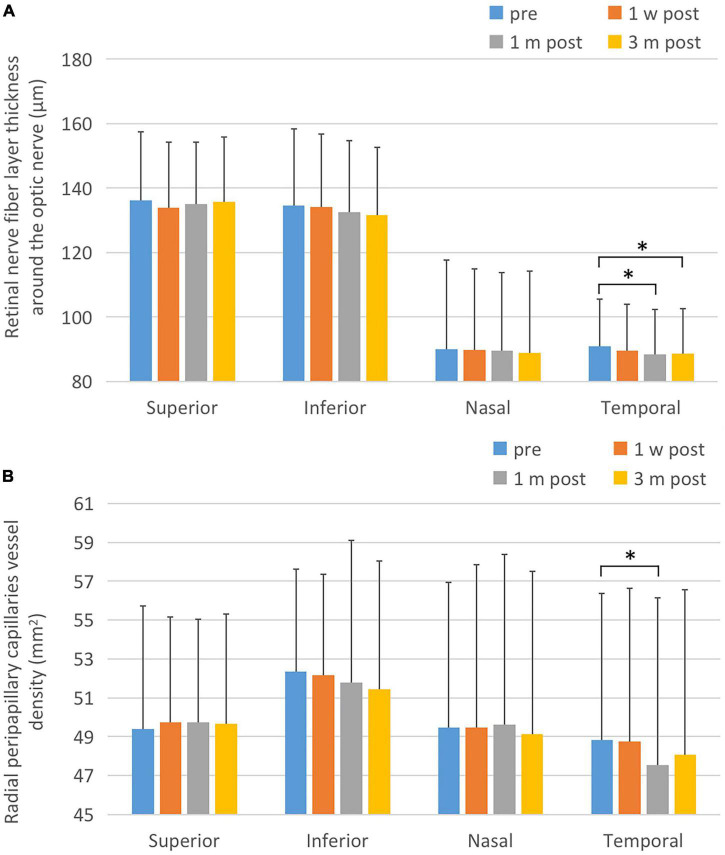
Changes of retinal nerve fiber layer thickness and peridisk capillaries after ICL implantation. **(A)** Postoperative RNFL changes. The temporal RNFL thickness at 1 and 3 months after surgery was significantly reduced compared to the corresponding pre-operative values. **(B)** Postoperative changes of capillary density of para-optic disk. One month after surgery, the postoperative peridisk capillary density of temporal side decreased significantly lower than that pre-operatively. The short black line shows that the data at the corresponding two time points were statistically different. *P < 0.05.

There was no statistically significant difference in the thickness at the upper, lower, and nasal side of the para-optic disk before and after surgery (F1 = 2.323, P1 = 0.089; F2 = 2.573, P2 = 0.055; F3 = 0.776, P3 = 0.508). The thickness of retinal nerve fiber layer (RNFL) at the temporal side of the para-optic disk at various time points was significantly different (*F* = 5.929, *P* = 0.001), and significantly decreased 1 month and 3 months after surgery compared with that pre-operatively (*P* = 0.002 and *P* = 0.032, respectively). There was no statistically significant difference in the blood flow density at the upper, lower, and nasal side of the para-optic disk before and after surgery (F1 = 0.216, P1 = 0.885; F2 = 1.046, P2 = 0.373; F3 = 0.259, P3 = 0.855). The temporal retinal blood flow density of the para-optic disk was significantly different at each time point (*F* = 3.003, *P* = 0.032), and significantly decreased 1 month after surgery compared with that before surgery (*P* = 0.043). As shown in Figure 4 and Table 4.

**TABLE 4:** Para-disk nerve fiber layer thickness and the blood flow density of radial peripapillary capillaries (RPC).

	Preoperative	1 week after surgery	1 month after surgery	3 months after surgery	*F*	*P*
Superior RNFL	136.2521.22	133.9120.30	135.1619.19	135.6620.21	2.323	0.076
Inferior RNFL	134.6123.85	134.0322.78	132.4522.34	131.6621.04	2.573	0.055
Nasal RNFL	90.0627.70	89.6725.25	89.5524.34	88.8725.27	0.168	0.892
Temporal RNFL	90.8814.64*^bc^*	89.5414.30	88.3613.95	88.7213.76	5.929	0.001
Superior RPC VD	49.406.34	49.755.40	49.735.30	49.645.65	0.216	0.885
Inferior RPC VD	52.345.26	52.165.18	51.787.32	51.456.58	1.046	0.373
Nasal RPC VD	49.467.46	49.488.37	49.618.78	49.128.40	0.259	0.855
Temporal RPC VD	48.827.56*^b^*	48.737.90	47.558.58	48.078.50	3.003	0.037

*RNFL, retinal nerve fiber layer; RPC, radial peripapillary capillaries; VD, vessel density. ^b^P < 0.05, values compared before surgery and 1 month after surgery.*

*^c^P < 0.05, values compared before surgery and 3 months after surgery.*

## Discussion

As the axis of the eye grows, high myopia can cause changes in retinal thickness, morphology, and retinal blood flow, which in turn leads to the occurrence of various retinal choroidal diseases, such as decreased in retinal blood flow and vascular stenosis (Al-Sheikh et al., 2017; Fan et al., 2017; Li et al., 2017). Gołêbiewska et al. (2019) observed the retinal blood vessel density of 96 children with myopia under 16 years of age and found that the FAZ of the myopic group was significantly higher than that of the control group. In addition, the longer the eye axis, the lower the density of superficial retinal blood vessels. A 21-month follow-up study of 16–28 year-old patients with myopia showed that the macular blood vessel density decreased with time, that its rate was faster than that of normal axis patients, Further, it was positively correlated with the axis of the eye (Shi et al., 2021). The above results indicate that the retinal blood vessel density in the macular area of patients with high myopia continue to change and may further lead to the occurrence of pathological myopia. After cataract surgery, the changes of retinal vascular density in patients with high myopia were more significant than those in normal axial populations, and the degree of change was related to the axis and IOP (Li et al., 2020). The peridiscal blood vessel density of patients with high myopia was also decreased (He et al., 2019), suggesting that the retinal blood vessels in patients with high myopia were more sensitive to intraocular surgery (Liu M. et al., 2021).

The superficial capillary network is mainly distributed in the nerve fiber layer and ganglion cell layer, while the deep capillary network is mainly distributed in the inner plexus layer and the inner core layer. In this study, it was found that although there was no significant difference of CRT between preoperative and postoperative, the thickness of the paracentral fovea retina in all quadrants was significantly increased 3 months after surgery, and the thickness of the nasal paracentral retina was significantly increased 3 months after surgery compared with that before surgery. In addition, the blood flow density postoperative in superficial and deep nasal paracentral fovea retina was significantly lower than that before surgery, suggesting that the decrease of GCL-IPL complex may be an important reason for the blood flow density change in superficial and deep layers after surgery (Hafner et al., 2019). At present, OCTA can only evaluate the retina of optic disk area within 4.5 mm × 4.5 mm. Within this range, the nasal retina gradually evolved from the posterior pole to the middle and peripheral part, accompanied by the reduction of capillary network layers and lack of physical support of nerve fibers, while the temporal retina still belongs to the posterior pole with abundant blood (de Carlo et al., 2015). This may also result in a more sensitive response of the nasal retina to environmental factors. Simultaneously, mechanical vitreous traction, destruction of the blood-retina barrier caused by the inner eye surgery, the imbalance of water flow between blood vessels and tissues, the accumulation of liquid in the outer plexiform layer in the macular area and the release of inflammatory cytokines such as prostaglandin caused by surgery are all important reasons for the changes (Gharbiya et al., 2013). Previous studies have shown that CRT increased significantly at 1 week, 1 month, and 3 months after ICL implantation, but the superficial and deep capillary density did not change obviously (Zhu et al., 2020). However, the study by Hu et al. (2021) found that retinal vascular density decreased significantly 1 week and 1 month after ICL implantation compared with that pre-operatively, and recovered to the pre-operative baseline level 3 months after surgery. Although the results of our study are not completely consistent with the previous conclusions, the changes in the characteristics of retinal blood vessels, such as the density of retinal microvessels and the thickness of retinal outer sublayer, especially the reduction of deep retinal capillary plexus, may increase the susceptibility to vascular-related diseases, and still suggest long-term follow-up. We should pay close attention to the possibility of the occurrence and development of pathological myopia in patients after ICL surgery.

Foveal avascular zone is a central foveal avascular area surrounded by interconnected capillaries, which is the most closely related to central vision and the prognosis. Past studies (Carpineto et al., 2016; Ng et al., 2016) have shown that the average physiological FAZ area of healthy participants was 200–400 μm^2^. Once lesion is involved, it can cause varying degrees of vision loss. In our study, the area of FAZ after ICL implantation was lower than that before surgery. Similarly, the area of FAZ showed a decreasing trend after phacoemulsification in patients with cataract, which may be related to surgical trauma and release of inflammatory factors. FAZ formation occurs before macular development during embryonic development, so it is essential for macular area development and fovea formation (Springer and Hendrickson, 2005). Wons et al. (2016) found that the area of FAZ increased in patients with retinal vein occlusion, and that the diameter of FAZ was negatively correlated with CDVA. Furthermore, the area of FAZ was also negatively correlated with visual acuity in patients with diabetic retinopathy (Balaratnasingam et al., 2016). In this study, CDVA was significantly improved after ICL implantation, which was not only related to the significant reduction of optical interference caused by glasses, but also possibly related to the reduction of FAZ.

In this study, the IOP increased obviously in the first week after surgery, and recovered to the pre-operative level in the first and third months after the operation. Fluctuations in IOP during intraocular surgery could significantly affect the fundus hemodynamics. For example, increased IOP during cataract surgery could reduce the blood flow rate of the central retinal artery, which in turn affects the blood perfusion of the inner retina (Saygili et al., 2012). Wang et al. (2020) performed laser peripheral iridotomy (LPI) in 97 patients with primary angle-closure suspects and observed the effect of changes in IOP on retinal blood flow and RPC. They found that the increase in IOP 1 h after LPI significantly reduced the retinal vascular and RPC density, and the higher the peak IOP, the more decreased in blood vessel density. The increase of superficial vascular density after cataract surgery in patients with high myopia was lower than that with low myopia (Benavente-Pérez et al., 2010; Wu et al., 2020), which might be related to the lower tolerance of retinal vessels to IOP fluctuations during intraocular surgery in patients with high myopia (Li et al., 2020). Hu et al. (2021) found that although IOP did not fluctuate after ICL, microvessel density still changed. This may be because the observation time point of IOP was 1 week, 1 month, and 3 months after surgery, and the fluctuation degree of IOP in the early postoperative period had not been observed. Meanwhile, as the retinal blood vessels are a microvascular system, the regulation ability of blood perfusion is low. These can lead to changes in microcirculation even if intraocular pressure was normal 1 week after surgery. Therefore, our study found that early postoperative increase in IOP may lead to decreased self-regulation of postoperative blood flow as well as insufficient filling and perfusion.

The RNFL thickness in patients with high myopia became thinner (Li et al., 2018; He et al., 2019) with an increase in diopter (Leung et al., 2006) or axial (Budenz et al., 2007). The reasons include thinning of the sclera and retina, narrowing and straightness of retinal blood vessels, hypoperfusion of the retina, changes in choroid and retinal microcirculation, occurrence of retinal ischemia and hypoxia, and degeneration of ganglion cell axons (Kamal Salah et al., 2015). Previous studies have shown that the decreased IOP in glaucoma patients increases perfusion of retinal microcirculation. Liu L. et al. (2021) found that the post-operative IOP decreased by an average of 5.3 mmHg, and that the perfusion of the retinal microcirculation of para-optic disk was significantly improved. Hence, changes in IOP could affect the perfusion of the retinal microcirculation of para-optic disk. This study showed that the temporal RNFL and the corresponding temporal RPC vessel density decreased after surgery, which may be related to the transient increase of IOP after ICL implantation, and suggested that the microcirculation of lacteal tract and RNFL are less tolerant to elevated IOP after ICL implantation. Chen et al. (2020) showed that only the decrease of temporal retinal vascular perfusion in the para-optic disk was significantly correlated with the degree of IOP, which was also consistent with this study.

The retina and optic nerves of highly myopic eyes are changed in comparison with normal eyes. ICL implantation might affect the microcirculation around the retina and optic disk. Therefore, more attention should be paid to the effects of IOP changes and postoperative inflammatory reactions on fundus blood flow, as well as postoperative visual function and prognosis. However, the sample size of this study was small, and the post-operative follow-up time was short. Therefore, it is necessary to continue to expand the sample size and conduct longer follow-up observation in the future, so as to further explore the long-term changes of retinal microcirculation after ICL, as well as the possible influence of fundus lesions and visual function.

## Data Availability Statement

The original contributions presented in the study are included in the article/supplementary material, further inquiries can be directed to the corresponding authors.

## Ethics Statement

This study complied with the Declaration of Helsinki and was reviewed and approved by the Medical Ethics Committee of The Affiliated Eye Hospital of Nanjing Medical University (Batch Number: 2019007). The patients/participants provided their written informed consent to participate in this study. Written informed consent was obtained from the individual(s) for the publication of any potentially identifiable images or data included in this article.

## Author Contributions

YX and KL carried out the studies and drafted the manuscript. WY participated in data collection. JW and JY participated in the study design and guidance. KL and XX performed the surgery. All authors read and approved the final manuscript.

## Conflict of Interest

The authors declare that the research was conducted in the absence of any commercial or financial relationships that could be construed as a potential conflict of interest.

## Publisher’s Note

All claims expressed in this article are solely those of the authors and do not necessarily represent those of their affiliated organizations, or those of the publisher, the editors and the reviewers. Any product that may be evaluated in this article, or claim that may be made by its manufacturer, is not guaranteed or endorsed by the publisher.

## Abbreviations

ICL: implantable collamer lens
OCTA: optical coherence tomography angiography
SE: spherical equivalent
IOP: intraocular pressure
FAZ: foveal avascular zone
UDVA: uncorrected distance visual acuity
CDVA: corrected distance visual acuity
ILM: inner limiting membrane
IPL: inner plexiform layer
CRT: central retinal thickness
FAZ-A: FAZ area
FAZ-CI: FAZ contour irregularity
RPC: radial peripapillary capillaries
RNFL: retinal nerve fiber layer
LPI: laser peripheral iridotomy
pre: pre-operative
post: post-operative
FVD: fovea vessel density
PVD: para-fovea vessel density
TVD: temporal vessel density
SVD: superior vessel density
NVD: nasal vessel density
IVD: inferior vessel density.
